# Assessing self-determined motivation for drinking alcohol via the Comprehensive Relative Autonomy Index for Drinking

**DOI:** 10.3389/fpsyg.2024.1354545

**Published:** 2025-01-08

**Authors:** Jimikaye Beck Courtney, Michael A. Russell, David E. Conroy

**Affiliations:** ^1^Prevention Research Center, Pennsylvania State University, State College, PA, United States; ^2^Health, Exercise, and Lifestyle Lab, Department of Exercise and Sport Science, University of North Carolina at Chapel Hill, Chapel Hill, NC, United States; ^3^Alcohol Habits in Daily Life Lab, Department of Biobehavioral Health, Pennsylvania State University, State College, PA, United States; ^4^Department of Kinesiology, Pennsylvania State University, State College, PA, United States; ^5^School of Kinesiology, University of Michigan, Ann Arbor, MI, United States

**Keywords:** alcohol drinking, motivation, Self-Determination Theory, confirmatory factor analysis, validation

## Abstract

**Introduction:**

Self-Determination Theory (SDT) examines human motivation in multiple domains; however, the only existing measure assessing SDT-informed behavioral regulations for drinking focuses on responsible drinker behaviors, rather than drinking *per se*, which is important given the alignment between SDT and harm reduction approaches to alcohol use. The aim of this study was to test the structural validity of the SDT-informed Comprehensive Relative Autonomy Index for Drinking (CRAI-Drinking) among college students.

**Methods:**

Participants included two convenience samples with a total of 630 adult drinkers (M_age_ = 21.5, 55% female, 88% undergraduates). Participants rated drinking behavioral regulations on the 24 original CRAI-Drinking items on a 5-point Likert Scale. Multi-dimensional scaling analyses and factor analyses were used to investigate the underlying autonomy continuum and factor structure of the CRAI-Drinking.

**Results:**

In Sample 1 (*n* = 274), multi-dimensional scaling analyses confirmed that CRAI-Drinking item and subscale order aligned with SDT's autonomy continuum. Confirmatory factor analyses supported a five factor, 19-item model of the CRAI-Drinking with factors for intrinsic, identified, positive introjected, external, and amotivation regulations (Cronbach's α: 0.68–0.85). In Sample 2 (*n* = 356), a confirmatory factor analysis confirmed that the 19-item model fit was comparable to Sample 1.

**Discussion:**

This study provides evidence for the structural validity of CRAI-Drinking scores for assessing SDT-based behavioral regulations for drinking in adults.

## 1 Introduction

Alcohol misuse is a significant public health concern in the U.S. (Hingson et al., 2017; Schulenberg et al., 2020), with over half of U.S. young adults using alcohol in the past month, 31% engaging in heavy episodic drinking (4+/5+ drinks for women/men) and 9% engaging in high intensity drinking (8+/10+ drinks for women/men) (Substance Abuse and Mental Health Services Administration Office of Applied Studies, 2021). Investigating individuals' unique reasons for drinking is useful for informing future interventions focused on promoting low-risk, responsible drinking behaviors, and harm reduction strategies. Cognitive, affective, and motivational factors impact drinking behaviors. For example, social cognition processes, explicit alcohol outcome expectancies (i.e., the consequences an individual expects to result from drinking), and implicit cognitions based on past experiences predict current and future drinking behaviors (Jajodia and Earleywine, 2003; Montes et al., 2017; Patrick et al., 2010; Wiers et al., 2002). These outcome expectancies are strongly linked to affect, and a person's affective experiences influence drinking behaviors, such that the effects of alcohol on affect may motivate drinking and drinking may impact affect (Dvorak et al., 2018). Drinking alcohol can enhance positive affect and decrease negative affect (Dvorak et al., 2018), thereby promoting positive outcome expectancies and reinforce coping, enhancing, intrinsic, and other motives for drinking (Cooper et al., 1992, 2008; Dvorak et al., 2018; Sher and Grekin, 2007; Wray et al., 2012). This study specifically focuses on drinking motives.

Drinking motives predict drinking behaviors and consequences and are essential for understanding alcohol use (Cooper, 1994; Cox, 1990; Cox and Klinger, 1988). One existing measure, the Drinking Motives Questionnaire (DMQ) assesses social, coping, conforming, and enhancing motives for drinking (Cooper, 1994; Cox and Klinger, 1988) and has been widely used to examine drinking motives as they relate to drinking contexts, behaviors, and consequences (Cooper, 1994; Gorka et al., 2017; Kuntsche et al., 2005; Kuntsche and Cooper, 2010; Kuntsche and Müller, 2012; Piasecki et al., 2014). DMQ drinking motives also mediate the effects of other psychosocial variables, such as alcohol use expectancies, intentions, social anxiety, and impulsivity, on drinking behaviors (Adams et al., 2012; Ham et al., 2009; Hasking et al., 2011; Kuntsche et al., 2007). However, researchers have recently proposed employing another theory of motivation, Self-Determination Theory (SDT), as an alternative framework for understanding drinking (Richards et al., 2021b; Sharma and Smith, 2011).

**Figure 1:**
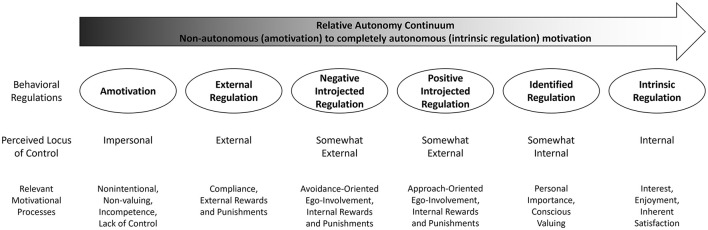
Self-Determination Theory—relative autonomy continuum, locus of control, and behavioral regulations. This figure shows Self-Determination Theory's conceptualization of human motivation. This shows the six motivational behavioral regulations ranging in their level of autonomy from low (non-autonomous amotivation) to high (completely autonomous intrinsic regulation), their perceived locus of control from impersonal (amotivation) to completely external (external regulation) to completely internal (intrinsic regulation), and the relevant motivational processes for each individual behavioral regulation.

Self-Determination Theory is a useful framework for understanding drinking because it accounts for several key psychological factors that predict drinking behaviors and outcomes by assessing behavioral regulations for engaging in a behavior that capture a broad spectrum of motives for consuming alcohol (Bhowmick et al., 2019; Chawla et al., 2009; Koski-Jännes, 1994; Lassi et al., 2019; Levesque et al., 2006; Peele and Brodsky, 2000; Ryan and Deci, 2000, 2017). SDT includes six behavioral regulations that are ordered along the relative autonomy continuum (RAC) from low to high levels of autonomy and external to internal locus of control (Figure 1; Ryan and Connell, 1989; Ryan and Deci, 2000, 2017; Sheldon et al., 2017). Intrinsic regulation is completely autonomous motivation in which people engage in a behavior because it is inherently interesting, stimulating, or enjoyable (Ryan and Connell, 1989; Ryan and Deci, 2000, 2017; Sheldon et al., 2017). Identified regulation is the most autonomous form of extrinsic motivation driven by personally valuing a behavior or its outcomes (Ryan and Connell, 1989; Ryan and Deci, 2000, 2017; Sheldon et al., 2017). Positive introjection describes engaging in a behavior to enhance internal feelings of self-worth (Assor et al., 2009; Sheldon et al., 2017). Negative introjection describes engaging in a behavior to avoid unpleasant internal self-conscious experiences such as loss of self-worth (Assor et al., 2009; Sheldon et al., 2017). External regulation is the least autonomous form of extrinsic motivation driven by the needs to avoid external punishment or to achieve rewards by complying with others' expectations (Ryan and Connell, 1989; Ryan and Deci, 2000, 2017; Sheldon et al., 2017). Amotivation is a completely non-autonomous regulation in which a person experiences no intentional motivation for their behavior (Ryan and Connell, 1989; Ryan and Deci, 2000, 2017; Sheldon et al., 2017).

Higher levels of more autonomous behavioral regulations (e.g., intrinsic, identified) support overall well-being and human flourishing (Ryan and Connell, 1989; Ryan and Deci, 2000, 2017). Research examining behavioral regulations and health behavior engagement indicates that higher levels of more autonomous regulations are associated with greater engagement in healthy behaviors and vice-versa (Chawla et al., 2009; Knee and Neighbors, 2002; Neighbors et al., 2003, 2004, 2007; Ng et al., 2012; Ryan et al., 2008; Ryan and Deci, 2007). Although few studies have examined self-determined behavioral regulations for drinking, the existing research indicates that lower levels of autonomous behavioral regulations are associated with heavier alcohol use (Knee and Neighbors, 2002; Neighbors et al., 2003, 2004, 2007). Other research examining the constructs underlying SDT's behavioral regulations, such as basic psychological needs (autonomy, competence, relatedness) and locus of control, has mixed findings. For example, several studies found that lower autonomy predicts increased drinking intensity and drinking for social approval (Chawla et al., 2009; Knee and Neighbors, 2002; Neighbors et al., 2003, 2004), whereas one study found that greater autonomy satisfaction corresponded with higher odds of drinking (Enns and Orpana, 2020). Similarly, moderate drinking may be associated with social benefits that support the need for relatedness (Peele and Brodsky, 2000; Sæther et al., 2019), but greater relatedness satisfaction may also correspond with lower odds of drinking (Enns and Orpana, 2020). Regarding locus of control, having a greater external or impersonal locus of control corresponds with greater alcohol use and hazardous drinking (Caudwell and Hagger, 2015; Chawla et al., 2009; Dukes et al., 2022; Koski-Jännes, 1994; Lassi et al., 2019), and people high on alcohol dependence experience a higher external locus of control (Bhowmick et al., 2019). Impersonal locus of control (i.e., amotivation) is associated with impulse control issues and maladaptive outcomes, suggesting it may be particularly relevant for research related to alcohol use disorders (Hofmann et al., 2009). Additional research is needed to understand how behavioral regulations relate to drinking behaviors and outcomes. However, such research is limited by the lack of a validated measure of self-determined behavioral regulations for drinking.

Currently, only one measure related to self-determined drinking behavioral regulations exists, the Treatment Self-Regulation Questionnaire (TSRQ), which assesses self-determined behavioral regulations for *responsible* drinking, such as being motivated to drink responsibly to take care of one's health or to get approval from others (Richards et al., 2020a,b). Conceptually, behavioral regulations for responsible drinking are not synonymous with behavioral regulations for drinking *per se*, as shown by research indicating that TSRQ behavioral regulations are not redundant for DMQ-assessed drinking motives and DMQ drinking motives account for more variance in alcohol-related outcomes than responsible drinking (Richards et al., 2021a). This lack of redundancy applies within the context of SDT, such that one person—Kathy—may drink due to enjoying the taste of alcohol (i.e., intrinsic regulation) but may drink responsibly due to pressure from others (i.e., external regulation). Conversely, another person—John—may drink due to external pressure from others but may be intrinsically motivated to drink responsibly. These distinctions are theoretically *and* practically important. From an intervention perspective, Kathy would benefit from an intervention targeting behavioral regulations for responsible drinking, whereas John would benefit from an intervention targeting behavioral regulations for drinking *per se*. Unfortunately, no validated measure of SDT-informed behavioral regulations for drinking currently exists, precluding researchers and interventionists from being able to distinguish between people like Kathy and John. Lastly, 37% of college students engage in risky drinking behaviors (Johnston et al., 2009), and behavioral regulations for responsible drinking are likely less relevant for that population than behavioral regulations for drinking in general.

The lack of a validated measure of self-determined behavioral regulations for drinking is problematic given SDT's alignment with psychological factors underlying alcohol use and its potential utility for alcohol use interventions. SDT is widely used in health behavior interventions, including alcohol use interventions (Sheeran et al., 2020), and has been highlighted as a promising theoretical framework for informing the development and refinement of alcohol use interventions (Richards et al., 2021b; Sharma and Smith, 2011; Sheeran et al., 2021). Targeting the psychological factors underlying SDT (e.g., autonomy, relatedness, intrinsic regulation) can effectively promote health behavior change, with interventions that increase more autonomous behavioral regulations resulting in small-to-medium positive changes in health behaviors (Ntoumanis et al., 2020; Ryan et al., 2008; Sheeran et al., 2021). Indeed, a meta-analysis of SDT interventions found that the interventions resulted in significant reductions in alcohol consumption (*d* = 0.26; Sheeran et al., 2020), and Dukes et al. (2022) cited the value of using SDT to inform substance use prevention and treatment interventions. Unfortunately, SDT is still widely understudied regarding alcohol use, and little is known about which behavioral regulations to target in interventions to reduce alcohol consumption. Developing a validated measure of SDT-informed behavioral regulations for drinking *per se* may provide researchers with a useful and comprehensive measure for investigating how the psychological factors underlying SDT relate to drinking behaviors and outcomes and has the potential for identifying relevant behavioral regulations that drive alcohol use targets for interventions. In the present research, we use two samples to examine the psychometric properties of a measure for assessing self-determined behavioral regulations for drinking.

Sheldon et al. (2017) developed the 24-item Comprehensive Relative Autonomy Index (CRAI) to create a common core of generic items that would enable researchers to assess the behavioral regulations underlying SDT's RAC across a variety of domains. The purpose of this study was to adapt Sheldon et al.'s (2017) CRAI to assess behavioral regulations for drinking underlying SDT's RAC. This will help provide initial psychometric evaluation of an SDT-based measure of behavioral regulations for drinking in general, rather than behavioral regulations for responsible drinking, among college students. Using two samples, we developed and cross-validated scores from the adapted survey—the Comprehensive Relative Autonomy Index for Drinking (CRAI-Drinking). We hypothesized that the order of CRAI-Drinking items/subscales would align with SDT's RAC and there would be six CRAI-Drinking subscales corresponding with a priori, theoretically-driven assignments for each item (Sheldon et al., 2017). Due to historical gender differences in alcohol use behaviors, we also tested measurement invariance of the CRAI-Drinking by gender (White, 2020; White et al., 2015).

## 2 Materials and methods

### 2.1 Participants and procedures

Participants included a convenience sample of adults 18 years or older (Sample 1) or 18–25 years (Sample 2) who consumed at least one drink/week. Study recruitment started November 19, 2020 and ended December 31, 2021. Sample 1 data were collected between November 2020 and May 2021. Of the 357 individuals who completed the online screening survey, 46 (12.9%) did not qualify due to insufficient alcohol use (< 1 drink/week), resulting in 306 (85.7%) qualified participants, 274 (89.5%) of whom completed the online study survey and were compensated with extra credit or entered to win one of two $50 gift cards. Sample 2 data were collected between October and December 2021. Of the 669 individuals who completed the online screening survey, 22.6% did not qualify due to insufficient alcohol use (< 1 drink/week), resulting in 515 (77.4%) qualified participants, 356 (69.1%) of whom completed the online study survey and were compensated with extra credit or entry to win 1 of 10 $30 gift cards. This study was approved by the university Institutional Review Board (Protocol #00016554) as an exempt protocol. Participants provided written implied informed consent to participate via the online survey platform (Research Electronic Data Capture).

### 2.2 Measures

#### 2.2.1 Comprehensive Relative Autonomy Index for Drinking items

The 24 CRAI-Drinking items were adapted from Sheldon et al.'s (2017) domain-agnostic version of the CRAI by modifying item prompts to refer specifically to drinking. Participants were asked: “Thinking of all the times you drink, how often would you say that you drink for each of the following reasons?” and rated each item on a 5-point Likert scale from (0) not true for me to (4) very true for me. Four items each assessed intrinsic, identified, positive introjected, negative introjected, and external regulations, and amotivation.

#### 2.2.2 Demographics

Demographic characteristics were assessed using self-reports of age, sex, ethnicity, race, education, work status, and student status.

### 2.3 Statistical analyses

Non-metric multidimensional scaling (NMDS) and Confirmatory Factor Analysis (CFA) were used to investigate structural validity. NMDS analyses were used to identify the spatial ordering of the CRAI-Drinking items/subscales to determine whether they followed the order specified by SDT's RAC, which assumes that the subscales follow this order from low to high levels of autonomy: amotivation, external regulation, negative introjected regulation, positive introjected regulation, identified regulation, and intrinsic motivation. NMDS was also employed to replicate Sheldon et al.'s (2017) study in which NMDS analyses were used when developing the domain-agnostic version of the CRAI. NMDS analyses identified whether the ordering of CRAI-Drinking items/subscales followed the assumptions of SDT's RAC based on the location of the items/subscales on the visual map (i.e., their x- and y-coordinates or angles; Hout et al., 2013; Sheldon et al., 2017). NMDS quantifies the similarity between items, and includes a visual map that conveys the spatial relationships among items, whereby similar items are more proximal to one another and dissimilar items are further apart (Hout et al., 2013). NMDS was used to test the simplex structure of the CRAI-Drinking, which would be represented by a semicircle on the map, and to examine the degree of similarity among items and subscales, as well as whether the ordering of items and subscales followed the order of SDT's RAC, both of which are reflected by item or subscale polar coordinates (i.e., angles; Hout et al., 2013; Sheldon et al., 2017). Item- and subscale-level correlation matrices were used to test one- and two-dimensional models. Model fit was assessed based on stress, which measures agreement between the model estimated and raw input data (i.e., correlation matrix), with lower stress values indicating better fit (Hout et al., 2013).

CFA was used to determine the factor structure (Brown, 2015; Kyriazos, 2018; Thompson, 1994, 2013). The NMDS subscale analyses informed the number of factors tested in the CFA, though additional CFA models with 1–6 factors were explored to confirm the best fitting model and to avoid confirmation bias (MacCallum and Austin, 2000; Supplementary Table B). Models were estimated using the weighted least squares mean and variance to account for the ordinal response options (Muthén et al., 1997). Absolute and relative fit indices used to assess model fit included Chi-square (χ^2^), comparative fit index (CFI), Tucker-Lewis index (TLI), root mean square error of approximation (RMSEA) and its confidence interval (CI), and the standardized root mean square residual (SMSR; Bentler, 1990; Bollen, 1989). Best practices for measurement in psychometrics require cross-validating the factor structure of the measure (Brown, 2015; Kline, 2016; Kyriazos, 2018; Thompson, 1994, 2013). Cross-validation safeguards the validity, reliability, and replicability of measurement in psychometrics by demonstrating the generalizability of factor structure across different samples (Brown, 2015; Kline, 2016; Kyriazos, 2018; Thompson, 1994, 2013). Therefore, Sample 2 was used to cross-validate the factor structure of the CRAI-Drinking. CFAs were conducted using the lavaan package using a full-information maximum likelihood estimator. Based on heuristics in the literature (Mundfrom et al., 2005), a hypothesized structure of six factors with four items per factor (Sheldon et al., 2017) and a moderate level of communality, a sample size of *N* = 300 was estimated to be sufficient to achieve good agreement (coefficient of congruence: K = 0.982) between sample and population solutions. Measurement invariance (e.g., configural, metric, scalar) was tested between genders. Configural invariance tested whether the overall factor structure fit well in both genders. Metric invariance tested whether the factor loadings were equivalent in both genders, and structural invariance tested whether the item intercepts were equivalent in both genders (Sass and Schmitt, 2013). Configural invariance was tested by fitting the final 5-factor model separately for males and females and comparing fit indices. Metric invariance was tested by constraining factor loadings to be equal and scalar invariance additionally constrained factor intercepts to be equal (Sass and Schmitt, 2013). Due to the potential oversensitivity of chi-square difference tests, models were compared using the criteria of a decrease in CFI ≥ 0.01 (Cheung and Rensvold, 2002) and an increase in RMSEA ≥ 0.015 (Chen, 2007) to be indicative of significantly worse model fit. Data were analyzed in R version 4.0.3 using the isoMDS, factoextra, and lavaan packages in R (Bollen, 1989; R Core Team, 2020; Rosseel, 2012).

## 3 Results

Sample 1 (*N* = 274) was 66.8% female, 91.6% non-Hispanic, 81.8% White, and 75.5% undergraduate students with a mean age of 23.0 ± 6.6 years. Sample 2 (*N* = 356) was 46.1% female, 89.9% non-Hispanic, 84.6% White, and 97.5% undergraduate students with a mean age of 20.4 ± 1.5 years. The combined sample (*N* = 630) was 55.1% female, 90.6% non-Hispanic, 83.3% White, and 88.1% undergraduate students with a mean age of 21.5 ± 4.7 years. Participants drank alcohol on 3.2 ± 1.5 days per week and consumed 8.5 ± 4.9 drinks per day. Supplementary Table A provides complete descriptive statistics for Sample 1 (*N* = 274), Sample 2 (*N* = 356), and the combined sample. Table 1 summarizes CRAI-Drinking item responses for Samples 1 and 2.

**Table 1:** Descriptive characteristics for the Comprehensive Relative Autonomy Index for Drinking for Sample 1 and Sample 2.

	**Sample 1 (*****n*** = **274)**				**Sample 2 (*****n*** = **356)**			
	**Range (Min–Max)**	**Mean** ±**SD**	**Skewness**	**Kurtosis**	**Range (Min–Max)**	**Mean** ±**SD**	**Skewness**	**Kurtosis**
**Intrinsic regulation**								
Q6/INT1: *Because drinking is fun*	0–4	3.0 ± 1.0	−0.86	3.53	0–4	3.3 ± 1.0	−1.56	5.44
Q12/INT2: *Because drinking is pleasurable*	0–4	2.7 ± 1.0	−0.66	3.09	0–4	3.1 ± 1.0	−1.01	3.61
Q18/INT3: *Because I enjoy drinking*	0–4	2.8 ± 1.1	−0.85	3.09	0–4	3.2 ± 1.0	−1.26	4.25
Q24/INT4: *Because drinking is satisfying to me*	0–4	1.7 ± 1.3	−0.01	1.84	0–4	2.3 ± 1.3	−0.37	1.97
**Identified regulation**								
Q5/IDENT1: *Because I personally choose to drink*	0–4	3.4 ± 0.9	−2.00	7.08	0–4	3.5 ± 0.9	−2.10	7.37
Q11/IDENT2: *Because I value the benefits of drinking*	0–4	1.0 ± 1.2	0.77	2.65	0–4	1.5 ± 1.4	0.39	1.87
Q17/IDENT3: *Because drinking is useful to me*	0–4	0.7 ± 1.0	1.27	3.70	0–4	1.2 ± 1.3	0.70	2.26
Q23/IDENT4: *Because drinking is personally important to me*	0–4	0.6 ± 0.8	1.48	4.88	0–4	0.9 ± 1.2	1.12	3.22
**Positive introjected regulation**								
Q4/POSREG1: *Because drinking boosts my self-esteem*	0–4	1.1 ± 1.1	0.77	2.80	0–4	1.9 ± 1.4	−0.00	1.72
Q10/POSREG2: *Because I want to feel confident in myself*	0–4	0.9 ± 1.1	1.01	3.03	0–4	1.5 ± 1.4	0.40	1.98
Q16/POSREG3: *Because drinking makes me feel more important*	0–4	0.4 ± 0.8	2.25	8.23	0–4	0.7 ± 1.0	1.57	4.63
Q22/POSREG4: *Because I want to feel good about myself*	0–4	0.8 ± 1.1	1.19	3.37	0–4	1.3 ± 1.3	0.57	2.02
**Negative introjected regulation**								
Q3/NEGREG1: *Because I would feel uncomfortable if I didn't drink*	0–4	0.8 ± 1.0	1.07	3.44	0–4	1.3 ± 1.3	0.55	2.07
Q9/NEGREG2: *Because I don't want to feel bad about myself*	0–4	0.4 ± 0.7	2.20	7.55	0–4	0.8 ± 1.2	1.50	4.24
Q15/NEGREG3: *Because I would feel embarrassed if I didn't drink*	0–4	0.4 ± 0.8	1.91	6.25	0–4	0.7 ± 1.0	1.50	4.55
Q21/NEGREG4: *Because I would feel awkward if I didn't drink*	0–4	0.9 ± 1.1	1.08	3.34	0–4	1.4 ± 1.4	0.52	2.03
**External regulation**								
Q2/EXTREG1: *Because important people will like me better if I drink*	0–3	0.4 ± 0.7	1.74	5.40	0–4	0.6 ± 1.0	1.58	4.70
Q8/EXTREG2: *Because my friends/family/partner say I should drink*	0–4	0.7 ± 1.0	1.36	3.84	0–4	0.7 ± 1.0	1.44	4.04
Q14/EXTREG3: *Because I feel under pressure from others to drink*	0–4	0.6 ± 0.9	1.36	4.23	0–4	0.7 ± 1.1	1.44	4.39
Q20/EXTREG4: *Because I want other people to think I'm fun*	0–4	0.9 ± 1.1	0.91	2.70	0–4	1.3 ± 1.3	0.60	2.26
**Amotivation**								
Q1/AMOT1: *I once had good reasons to drink, now I don't*	0–4	0.9 ± 1.1	0.98	3.11	0–4	1.0 ± 1.1	0.85	2.85
Q7/AMOT2: *Honestly, I don't know why I drink*	0–4	0.8 ± 1.1	1.25	3.94	0–4	1.1 ± 1.2	0.84	2.91
Q13/AMOT3: *I used to know why I drink, but I don't anymore*	0–4	0.3 ± 0.7	2.57	9.85	0–4	0.5 ± 0.9	1.72	5.52
Q19/AMOT4: *I am not sure why I drink, I wonder whether I should continue*	0–4	0.6 ± 1.0	1.73	5.49	0–4	0.8 ± 1.2	1.34	3.78

Min, minimum; Max, maximum; SD, standard deviation; INT, intrinsic regulation; IDENT, identified regulation; POSREG, positive introjected regulation; NEGREG, negative introjected regulation; EXTREG, external regulation; AMOT, amotivation.

**Figure 2:**
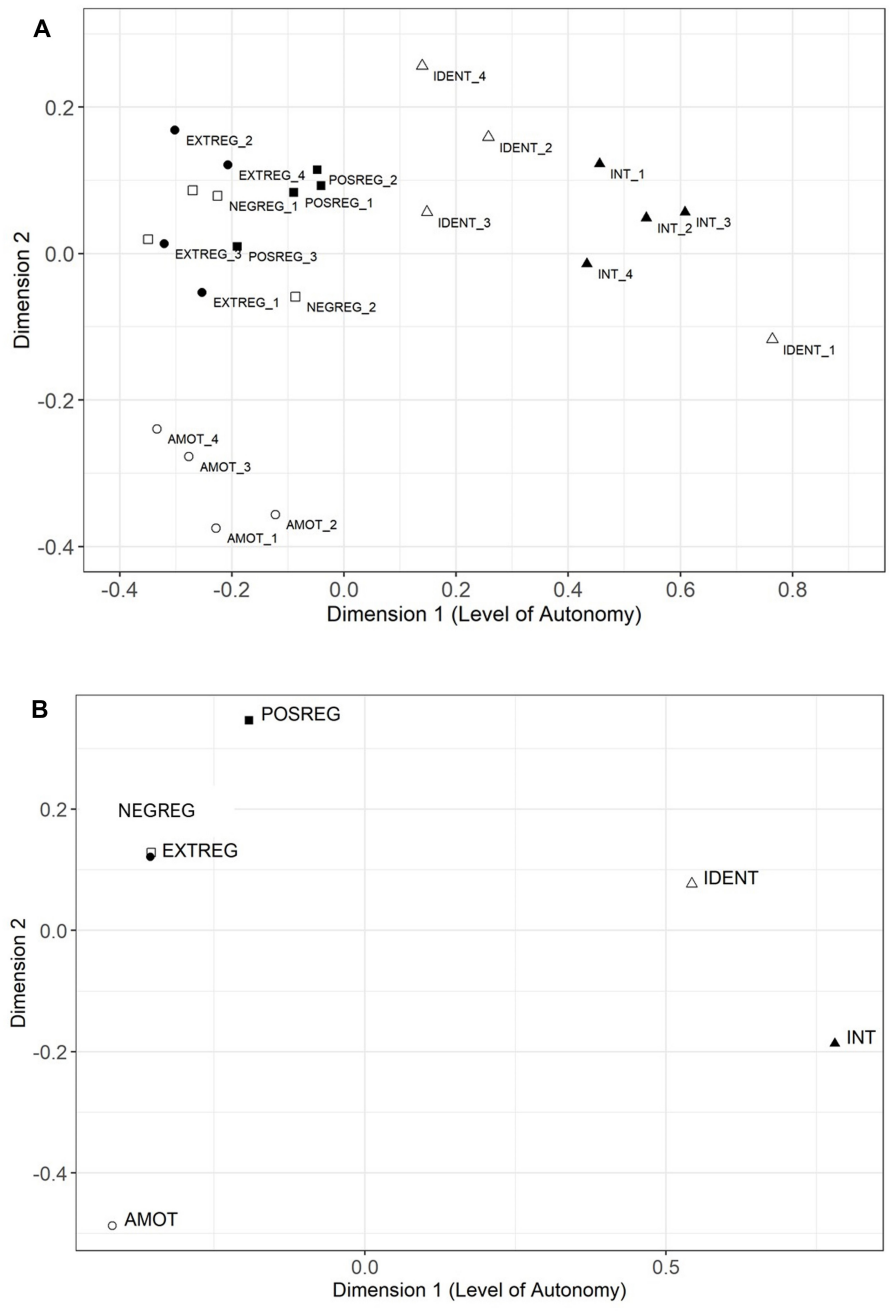
Results of non-metric multidimensional scaling analysis at the item **(A)** and scale **(B)** levels. This figure shows the results of the NMDS analyses of the individual CRAI-Drinking items (1A) and subscales (1B). The analyses revealed a two-dimensional structure for the items and subscales, which are represented by Dimensions 1 and 2 **(A, B)**. Dimension 1 can be inferred as representing the level of autonomy characterized by each item (2A) or subscale (2B). For example, in 2A, item AMOT_3 reflects a low level of autonomy, whereas item INT_2 reflects a high level of autonomy. In 2B, subscale AMOT (representing the four amotivation items) reflects a low level of autonomy, whereas subscale INT (representing the four intrinsic regulation items) reflects a high level of autonomy. As seen in 2B, the external regulation and negative introjected regulation subscales are located in essentially the same place on the map, indicating they are highly similar to one another.

The NMDS item-level analysis of Sample 1 data indicated that the two-dimensional model fit better (stress = 0.110) than the one-dimensional model (stress = 0.166). The ordering of items predominantly aligned with SDT's RAC (Figure 2A), although identified regulation item 1 (angle = 1.72) had a higher angle (e.g., greater autonomy) than the intrinsic regulation items (angles = 1.31 to 1.60). The subscale-level analysis showed that the ordering of subscales reflected SDT's RAC (Figure 2B). However, external and negative introjected regulation overlapped on the map (external regulation angle = −1.24, negative introjected angle = −1.22), indicating that they were highly similar to one another and essentially represented the same factor. Based on these findings, we dropped the negative introjected items and retained the external regulation items to preserve a parallel structure with the TSRQ (Richards et al., 2020a). The positive introjected items ensured that introjection would still be uniquely represented in the measure. Item 1 for identified regulation did not follow SDT's RAC based on NMDS analysis and was dropped from subsequent models. Of the 24 original CRAI-Drinking items, 19 were retained that represented five factors: intrinsic (4 items), identified (3 items), positive introjected (4 items), and external (4 items) regulations, and amotivation (non-regulation; 4 items).

Figure 2 shows the results of the NMDS analyses of the individual CRAI-Drinking items (1A) and subscales (1B). The analyses revealed a two-dimensional structure for the items and subscales, which are represented by Dimensions 1 and 2 in Figures 2A, B. Dimension 1 can be inferred as representing the level of autonomy characterized by each item (2A) or subscale (2B). For example, in 2A, item AMOT_3 reflects a low level of autonomy, whereas item INT_2 reflects a high level of autonomy. In 2B, subscale AMOT (representing the four amotivation items) reflects a low level of autonomy, whereas subscale INT (representing the four intrinsic regulation items) reflects a high level of autonomy. As seen in 2B, the external regulation and negative introjected regulation subscales are located in essentially the same place on the map, indicating they are highly similar to one another.

Results of the NMDS analyses informed the subsequent CFA examining 19 items loading on to five factors. CFA showed that the five-factor model demonstrated good model fit, χ^2^ (142) = 165.463 (*p* = 0.087), CFI = 0.991, TLI = 0.990, RMSEA = 0.025 (95% CI [0.000, 0.040]), and SMSR = 0.061. All retained items loaded significantly onto their factors (*p* < 0.001) with standardized factor loadings ranging from 0.56 to 0.81. Reliability estimates were high for each factor based on Cronbach's alpha values of 0.82 for intrinsic, 0.66 for identified, 0.85 for positive introjected, and 0.80 for external regulations, and 0.76 for amotivation. As expected, correlations were stronger between factors that shared similar levels of autonomy (e.g., positive introjected and external regulations: *r* = 0.65) and weaker for factors that had less similar levels of autonomy (e.g., intrinsic and external regulations: *r* = 0.03; intrinsic regulation and amotivation: *r* = −0.02; see Table 2 for the correlation matrix for the CRAI-Drinking factors). Due to Sheldon et al.'s (2017) original CRAI including six factors, we tested a six-factor model, as well as four additional theoretically-plausible measurement models to avoid confirmation bias (MacCallum and Austin, 2000). Supplementary Table B shows the model fit for these additional models. As expected, the hypothesized six-factor model did not converge due to a perfect correlation between the negative introjected and external regulation factors. Additionally, the five-factor model had better fit indices than alternative models with one to four factors, as demonstrated by a lower χ^2^ value that was not statistically significant, higher CFI and TLI values, and lower RMSEA and SMSR values than all other models (Bentler, 1990; Bollen, 1989); therefore, the five-factor model was selected as the model of best fit.

**Table 2:** Correlation coefficient matrix of mean scores for all factors for the Comprehensive Relative Autonomy Index for Drinking^a^.

		**CRAI-Drinking factors**			
		**INT (1)**	**IDENT (2)**	**POSREG (3)**	**EXTREG (4)**
CRAI-Drinking factors	INT (1)				
	IDENT (2)	0.46^*^			
	POSREG (3)	0.26^*^	0.53^*^		
	EXTREG (4)	0.06	0.37^*^	0.66^*^	
	AMOT (5)	−0.03	0.22^*^	0.34^*^	0.57^*^

^*^p < 0.005. Bonferroni adjustment critical *p* for 10 tests = 0.005; critical *r* = 0.19. CRAI-Drinking, Comprehensive Relative Autonomy Index for Drinking; DMQ, Drinking Motives Questionnaire; INT, intrinsic regulation; IDENT, identified regulation; POSREG, positive introjected regulation; NEGREG, negative introjected regulation; EXTREG, external regulation; AMOT, amotivation. ^a^N = 231.

As shown in Table 3, cross-validation of the five-factor model in Sample 2 revealed similar and good model fit indices to those found in Sample 1. In Sample 2 all items loaded significantly onto their factors (*p* < 0.001), and the standardized factor loadings and reliability estimates were similar to those in Sample 1 (e.g., factor loadings ranged from 0.41 to 0.83), supporting the five-factor solution. Supplementary Table C includes the variance/covariance matrix for all CRAI-Drinking items for Samples 1 and 2.

**Table 3:** Goodness-of-fit indexes for the 5-factor model between Samples 1 and 2 and testing measurement invariance by gender.

	**Overall model fit statistics**									
**Sample** ^a^	χ^2^ (*df*)	***p***	**CFI**	**TLI**	**RMSEA (95% CI)**	**SMSR**						
Sample 1	165.463 (142)	0.087	0.991	0.990	0.025 (0.000, 0.040)	0.061						
Sample 2	246.022 (142)	< 0.001	0.976	0.971	0.048 (0.038, 0.058)	0.067						
**Gender** ^b^	χ^2^ (*df*)	***p***	**CFI**	**TLI**	**RMSEA (95% CI)**	**SMSR**						
Males (*n* = 283)	146.348 (142)	0.384	0.999	0.998	0.011 (0.000, 0.032)	0.060						
Females (*n* = 347)	272.535 (142)	< 0.001	0.969	0.962	0.053 (0.043, 0.062)	0.069						
**Invariance Testing** ^c^	χ^2^ (*df*)	***p***	**CFI**	**TLI**	**RMSEA (95% CI)**	**SMSR**		Δχ^2^	*p* ^b^	Δ*CFI*	Δ*TLI*	Δ*RMSEA*
1. Configural Model	418.884 (284)	< 0.001	0.982	0.978	0.040 (0.032, 0.048)	0.065						
2. Metric Model	483.971 (298)	< 0.001	0.975	0.971	0.046 (0.039, 0.054)	0.070	1 vs. 2	65.087	0.005	−0.007	−0.007	0.006
3. Scalar Model	498.038 (312)	< 0.001	0.975	0.973	0.045 (0.04, 0.05)	0.071	2 vs. 3	14.067	0.018	0.000	0.002	−0.001

χ^2^, Chi-square; df, degrees of freedom; *p*, p-value; CFI, Comparative Fit Index; TLI, Tucker Lewis Index; RMSEA, Root Mean Square Error of Approximation; CI, Confidence Interval; SMSR, Standardized root Mean Square Residual. ^a^Models were estimated using the weighted least mean square and variance (WLMSV) estimator in lavaan, which does not allow missing data (Muthén et al., 1997; Rosseel, 2012). The analytic sample for Sample 1 was n = 264. The analytic sample for Sample 2 was n =322. ^b^The achieve a sufficient sample size these models are based on the combined sample of males and females. Models were estimated using the WLMSV estimator in lavaan. The analytic sample size for males was n = 256. The analytic sample size for females was n = 330. ^c^Invariance testing was conducted on the combined sample (N = 630) to support a sufficient samples size. ^b^Chi-square difference tests are often oversensitive. We report these values for transparency but follow recommendations for examining change in CFI and change in RMSEA to assess significant changes in model fit (Chen, 2007; Cheung and Rensvold, 2002).

*Post-hoc* analyses testing invariance by gender used the combined sample (males: *n* = 283, females: *n* = 347). As shown in Table 3, fit indices for the final five-factor model were slightly better for males, but both genders demonstrated good model fit, and all items loaded significantly onto their factors (*p* < 0.001), supporting configural invariance, meaning that the five-factor structure fit well in both genders. Neither the metric model nor the scalar model demonstrated significant decrements in fit compared to the configural and metric models, respectively, based on small changes in the CFI and RMSEA that were within the acceptable range to support metric and scalar invariance across genders. Metric invariance indicated that the factor loadings were equivalent in both genders, and scalar invariance indicated that the item intercepts were equivalent in both genders.

## 4 Discussion

This study provides initial support for the structural validity of CRAI-Drinking scores, a new measure of behavioral regulations for drinking adapted from Sheldon et al.'s (2017) CRAI. The CRAI-Drinking distinguished between five behavioral regulations for drinking, including intrinsic, identified, positive introjected, and external regulations, and amotivation. This initial testing of the CRAI-Drinking fills a gap by providing a novel measure of SDT-based behavioral regulations for general drinking behaviors among young adults. This contributes to the literature, as the only previously existing SDT-informed measure of drinking was the TSRQ, which assesses self-determined behavioral regulations for responsible drinking (Richards et al., 2020a,b). General motives for drinking are conceptually and practically distinct from motives for responsible drinking and account for more variance in drinking outcomes (Richards et al., 2021a). Establishing the CRAI-Drinking as a valid measure of self-determined behavioral regulations for drinking *per se* contributes to the literature by providing researchers with a comprehensive measure of self-determined behavioral regulations for drinking that will support future research examining how the psychological factors underlying SDT relate to drinking behaviors, outcomes, and alcohol use disorder and may help in identifying relevant psychological targets for developing, mechanistically evaluating, or refining interventions.

The CRAI-Drinking model demonstrated good structural validity; however, in contrast to Sheldon et al.'s (2017) original CRAI measure, which was developed as a common core of domain-agnostic items that would enable researchers to assess SDT's individual behavioral regulations across a variety of domains, external and negative introjected regulation were not distinct when considering behavioral regulations for drinking in these samples. After removing five items, a five-factor model with factors for intrinsic, identified, positive introjected, and external regulation and amotivation demonstrated good fit across both samples. The correlations between the five factors reflected SDT's RAC, such that behavioral regulations characterized by more similar levels of autonomy demonstrated stronger correlations with one another than regulations that were less similar to one another (Ryan and Connell, 1989; Ryan and Deci, 2000, 2017; Sheldon et al., 2017).

Contrary to our hypothesis and original CRAI Sheldon et al.'s (2017), external and negative introjected regulation were not distinct from one another. Previous research indicated that external regulations involving social incentives, such as those assessed by the CRAI-Drinking, were closely related to introjection (Gagné et al., 2015; Roth et al., 2006). It is possible that the influence of social norms on drinking behaviors and motives closed the gap between external and negative introjected regulation, such that they were indistinguishable with regard to drinking. Social motives and norms play a particularly important role in drinking among college students (Cooper, 1994; Foster et al., 2015; Sudhinaraset et al., 2016; Patrick et al., 2017), which could have affected our findings given the large proportion of undergraduate students in our sample. Future studies should investigate whether external regulation and negative introjection represent distinct behavioral regulations for drinking among samples with a larger proportion of non-undergraduate student drinkers.

Among all of the behavioral regulations assessed via the CRAI-Drinking, amotivation is distinct because it is a completely non-regulated behavioral regulation characterized by an impersonal locus of control and unknown or unclear reasons for drinking (Ryan and Connell, 1989; Ryan and Deci, 2000, 2017; Sheldon et al., 2017). The CRAI-Drinking is the first measure assessing amotivation for drinking *per se* and is unique from existing drinking motives measures in accounting for locus of control (Ryan and Connell, 1989; Ryan and Deci, 2000, 2017; Sheldon et al., 2017). This is important given that having an external or impersonal locus of control corresponds with maladaptive behavioral outcomes, including increased alcohol use and temptation to use alcohol (Dukes et al., 2022), worse alcohol use behaviors among people with and without alcohol use disorder (Bhowmick et al., 2019; Caudwell and Hagger, 2015; Chawla et al., 2009; Dukes et al., 2022; Lassi et al., 2019), and worse post-recovery drinking behaviors among recovering alcoholics (Koski-Jännes, 1994). Impersonal locus of control is also related to impulse control issues, suggesting it may be particularly relevant for studying alcohol use disorders (Hofmann et al., 2009). Lastly, Dukes et al. (2022) highlight to importance of targeting amotivation/impersonal locus of control in substance use interventions to empower individuals to feel a greater sense of control and to support better substance use behaviors and outcomes. As such, amotivation is a unique behavioral regulation that likely requires greater attention, and the development of the CRAI-Drinking supports future research and interventions investigating amotivation related to alcohol use.

The five-factor model of CRAI-Drinking scores demonstrated configural, metric, and scalar invariance between genders. This is important given historical differences in alcohol consumption between males and females (White, 2020; White et al., 2015). Configural invariance indicates that the number of factors and the specific pattern of items for each factor are the same for males and females (Brown, 2015), indicating that both genders interpret the items similarly, which is a necessary prerequisite for examining gender differences in drinking behavioral regulations. Achieving metric and scalar invariance supports the ability to compare latent means between groups (Brown, 2015), which is valuable for future research examining gender similarities or differences in self-determined behavioral regulations for drinking.

The study was limited to two convenience samples of young adults who were predominantly White, non-Hispanic undergraduate students. More diverse samples should be tested to examine factor structure and before generalizing inferences about score meaning to other groups, including people with alcohol use disorders or neuropsychiatric disorders. CRAI-Drinking response options were modified from the original survey to align with other SDT-based measures (Markland and Tobin, 2004), which may have affected findings.

Data collection overlapped with the COVID-19 pandemic. Findings from the Monitoring the Future study indicated that more young adults reported drinking to relax/relieve tension or because of boredom and more reported drinking alone or at home from April to November 2020 compared to the 5 years prior to the pandemic (Patrick et al., 2022). Young adults also had significantly lower prevalence rates of past 30-day drinking and binge drinking, though young adults who did drink reported significantly higher frequency of past 30-day drinking and binge drinking from April to November 2020 (Patrick et al., 2022). We found that Sample 1, whose data were collected after lockdowns ceased but prior to vaccines becoming widely available and when many undergraduate universities were holding remote (Fall 2020) and/or a combination of remote, hybrid, and in-person classes (Fall 2021), reported significantly less drinking and lower levels of all SDT-behavioral regulations for drinking compared to Sample 2. Sample 2 data were collected after vaccines became widely available and when most universities were holding in-person classes (Fall 2021). However, these differences between the samples did not affect the structural validity of the CRAI-Drinking, as the 5-factor model showed good fit in both samples. This suggests that, despite potential effects of the COVID-19 pandemic on drinking reasons and/or behaviors, the CRAI-Drinking is a structurally valid measure for assessing SDT's behavioral regulations for drinking.

The negative introjection items assessed avoiding self-conscious experiences (e.g., embarrassment) and loss of status, rather than guilt or shame. This is consistent with other SDT-measures of negative introjection but may have impacted our findings. Studies testing additional negative introjection items directly pertaining to other unpleasant anticipatory emotions (e.g., guilt, shame, anxiety) would be worthwhile. Although we found measurement invariance by gender, additional research examining measurement invariance by other sociodemographic characteristics (e.g., student status, age) and longitudinal invariance is also warranted. Studies employing cognitive interviewing or investigating response processes would be useful for providing additional support for substantive validity and interpretation of CRAI-Drinking items and responses (Boness and Sher, 2020; Hubley and Zumbo, 2017).

This study contributes to the literature by providing initial support for the use of the CRAI-Drinking as a measure of self-determined behavioral regulations for general drinking behavior. The 19-item CRAI-Drinking provides scores for five behavioral regulations for alcohol use: intrinsic, identified, positive introjected, and external regulations and amotivation and shows good evidence of internal scale validity and measurement invariance between genders. The CRAI-Drinking has the potential to provide useful information to researchers regarding how self-determined behavioral regulations and key psychological constructs underlying SDT are related to drinking behaviors and outcomes to inform general knowledge and to identify targets for behavioral interventions. Future studies should investigate the validity of the CRAI-Drinking for predicting drinking behaviors and consequences.

## Data Availability

The raw data supporting the conclusions of this article will be made available by the authors, without undue reservation.
