# Supplementary material for: Assessing self-determined motivation for drinking alcohol via the Comprehensive Relative Autonomy Index for Drinking
Source: Front Psychol. 2025 Jan 8;15:1354545. doi: 10.3389/fpsyg.2024.1354545 (PMC11751038; doi:10.3389/fpsyg.2024.1354545)
Supplement: Supplementary file 1 [file Table_1.docx]

Supplementary Material

Assessing self-determined motivations for drinking alcohol via the Comprehensive Relative Autonomy Index for Drinking

Jimikaye Beck Courtney^*^, Michael A Russell, David E Conroy

*** Correspondence:** Corresponding Author: jimikaye@unc.edu

# Supplemental Table A

| *Sample characteristics* | | | |
| --- | --- | --- | --- |
| Demographic Characteristics | Sample 1 (N=274) | Sample 2 (N=356) | Combined Sample (N=630) |
| Age (Mean ± SD) | 23.0 ± 6.6 | 20.4 ± 1.5 | 21.5 ± 4.7 |
| Female (n (%)) | 183 (66.8) | 164 (46.1) | 347 (55.1) |
| Race (n (%)) ^a^ |  |  |  |
| White | 224 (81.8) | 301 (84.6) | 525 (83.3) |
| Asian | 25 (9.1) | 23 (6.5) | 48 (7.6) |
| Black | 11 (4.0) | 3 (0.8) | 14 (2.2) |
| American Indian/Alaskan Native | 1 (0.4) | 1 (0.3) | 2 (0.3) |
| Other | 1 (0.4) | 4 (1.1) | 5 (0.8) |
| Mixed | 9 (3.3) | 18 (5.1) | 27 (4.3) |
| Education level (n (%)) ^b^ |  |  |  |
| High school degree or equivalent | 93 (33.9) | 134 (52.3)* | 227 (36.0) |
| Some college or AA degree | 118 (43.1) | 203 (57.0)* | 321 (50.8) |
| Bachelor’s Degree | 26 (7.0) | 17 (4.8)* | 43 (6.8) |
| Post-Graduate Degree | 37 (13.5) | 1 (0.3)* | 38 (6.0) |
| Work Status (n (%)) ^c^ |  |  |  |
| Full-time | 40 (14.6) | 2 (0.6)* | 42 (6.7) |
| Part-time | 2 (0.7) | 0 (0.0) | 2 (0.3) |
| Unemployed/Looking for Work | 1 (0.4) | 0 (0.0) | 1 (0.2) |
| Student, not working | 121 (44.2) | 155 (43.5) | 276 (43.8) |
| Student, working | 108 (39.4) | 198 (55.6)* | 306 (48.6) |
| Other work status | 2 (0.7) | 0 (0.0) | 2 (0.3) |
| Student Type (*n* (%)) ^d^ |  |  |  |
| Undergraduate Student | 208 (75.5) | 347 (97.5)* | 555 (88.1) |
| Post-Graduate Student | 17 (6.2) | 7 (2.0)* | 24 (3.8) |
| Non-Student | 43 (15.7) | 2 (0.6)* | 45 (7.1) |
|  |  |  |  |
| Alcohol Use Characteristics | Mean ± SD | Mean ± SD | Mean ± SD |
| Alcohol Use – Typical Week |  |  |  |
| Total Drinks per Week ^e^ | 11.2 ± 8.4 | 18.2 ± 13.1 | 15.2 ± 11.8 |
| Days per Week | 2.9 ± 1.4 | 3.5 ± 1.6 | 3.2 ± 1.5 |
| Drinks per Day | 3.9 ± 2.3 | 5.1 ± 2.8 | 8.5 ± 4.9 |
|  |  |  |  |
| CRAI-Drinking ^f^ |  |  |  |
| Intrinsic Regulation | 2.6 ± 0.9 | 2.9 ± 0.9* | 2.8 ± 0.9 |
| Identified Regulation | 0.8 ± 0.8 | 1.2 ± 1.1* | 1.0 ± 1.0 |
| Positive Introjected Regulation | 0.8 ± 0.8 | 1.3 ± 1.1* | 1.1 ± 1.0 |
| External Regulation | 0.7 ± 0.7 | 0.8 ± 0.8* | 0.9 ± 0.8 |
| Amotivation | 0.6 ± 0.7 | 0.9 ± 0.7* | 0.8 ± 0.8 |
|  |  |  |  |
| Drinking Motives Questionnaire ^g^ |  |  |  |
| Social | 3.2 ± 1.0 | 3.6 ± 1.0* | 3.4 ± 1.0 |
| Coping | 2.0 ± 0.9 | 2.4 ± 1.1* | 2.2 ± 1.0 |
| Enhancing | 2.8 ± 0.9 | 3.3 ± 1.0* | 3.0 ± 1.0 |
| Conforming | 1.5 ± 0.7 | 1.7 ± 0.9 | 1.6 ± 0.8 |
| Notes: **p* < .01 for difference between samples based on Chi-square test, *t*-test, or Wilcoxon test. SD = standard deviation; AA = Associate’s Degree; Min = minimum; Max = maximum; 1QR = first quartile; 3QR = third quartile. CRAI-Drinking = Comprehensive Relative Autonomy Index for Drinking. ^a^ Missing data: Sample 1 = 2; Sample 2 = 6. ^b^ Missing data: Sample 1 = 0; Sample 2 = 1. Some college or AA degree includes some college education, Associate’s degree, or trade school. Post-Graduate Degree includes Master’s degree, Doctoral degree, and professional degree (e.g., medical doctor). ^c^ Missing data: Sample 1 = 0; Sample 2 = 1. Student, not working includes undergraduate and graduate students who are not working any part-time or full-time job. Student, working includes undergraduate and graduate students who are also working a part-time or full-time job. ^d^ Missing data: Sample 1 = 5; Sample 2 = 1. Post-Graduate Student includes graduate and professional students. ^e^ Sum of drinks per day for typical week, with outliers (*n* = 11) winsorized to 3 SDs above the mean. ^f^ Combined sample Cronbach’s alpha (α) and 95% Confidence Intervals (CI). Intrinsic regulation α = 0.82 (95% CI: 0.78, 0.84). Identified regulation α = 0.72 (95% CI: 0.67, 0.76) (excludes IDENT_1 item). Positive introjected regulation α = 0.86 (95% CI: 0.83, 0.87). External regulation α = 0.75 (0.71, 0.78). Amotivation α = 0.78 (0.74, 0.82). ^h^ Combined sample Cronbach’s α and CI. Social motives α = 0.87 (95% CI: 0.85, 0.89). Coping motives α = 0.87 (95% CI: 0.85, 0.89). Enhancing motives α = 0.84 (0.82, 0.86). Conforming motives α = 0.85 (0.82, 0.88). | | | |

# Supplemental Table B

| *Comparing goodness of fit indexes for different theoretically plausible models in Sample 1 (n = 274)* ^a,b^ | | | | | | | |
| --- | --- | --- | --- | --- | --- | --- | --- |
|  | | Model Fit Statistics | | | | | |
| Model | Descriptions of Models Tested | $X^{2}$ (df) | *p* | CFI | TLI | RMSEA (95% CI) | SMSR |
| 1 | All items load onto single factor | 1080.622 (152) | <.001 | 0.52 | 0.46 | 0.16 (0.15, 0.17) | 0.14 |
| 2 | (1) Motivation Factor (all non-AMOT items); (2) Amotivation (all AMOT items) | 920.860 (151) | <.001 | 0.61 | 0.55 | 0.15 (0.14, 0.16) | 0.14 |
| 3a | (1) Intrinsic Regulation (all INT items); (2) Extrinsic Motivation (all EXTREG, POSREG, and IDENT items); (3) Amotivation (all AMOT items) | 545.173 (149) | <.001 | 0.80 | 0.77 | 0.11 (0.10, 0.12) | 0.10 |
| 3b | (1) Autonomous Motivation (all IDENT and INT items); (2) Controlled Motivation (all EXTREG, POSREG items); (3) Amotivation (all AMOT items) | 591.573 (149) | <.001 | 0.77 | 0.74 | 0.11 (0.10, 0.12) | 0.12 |
| 4 | (1) Intrinsic Regulation (all INT items); (2) Identified Regulation (all IDENT items); (3) Controlled Motivation (all EXTREG, POSREG items); (4) Amotivation (all AMOT items) | 470.158 (146) | <.001 | 0.84 | 0.81 | 0.10 (0.09, 0.11) | 0.08 |
| **5** | **(1) Intrinsic Regulation (all INT items); (2) Identified Regulation (IDENT items 2, 3, 4); (3) Positive Introjected Regulation (all POSREG items); (4) External Regulation (all EXTREG items); (5) Amotivation (all AMOT items)** | **384.234 (142)** | **<.001** | **0.89** | **0.87** | **0.08 (0.07, 0.09)** | **0.06** |
| Notes: $X^{2}$ = Chi-square; df = degrees of freedom; *p* = p-value; CFI = Comparative Fit Index, TLI = Tucker Lewis Index; RMSEA = Root Mean Square Error of Approximation; CI = Confidence Interval; SMSR = Standardized root Mean Square Residual. The bolded model was the final model selected for use in subsequent analyses. The **bolded row** indicates the 5-factor model and the model of best fit. ^a^ Models do not include NEGREG items 1 – 4 or IDENT1 item due to the results from the nonmetric multidimensional scaling analysis. ^b^ The hypothesized 6-factor model is not shown because the model failed due to a perfect correlation between the external regulation and negative introjected regulation factors. | | | | | | | |

# Supplemental Table C

| *Covariance matrix for all items on the original Comprehensive Relative Autonomy Index for Drinking for Sample 1 and Sample 2 ^a^* | | | | | | | | | | | | | | | | | | | | | | | | |
| --- | --- | --- | --- | --- | --- | --- | --- | --- | --- | --- | --- | --- | --- | --- | --- | --- | --- | --- | --- | --- | --- | --- | --- | --- |
|  | Q6 | Q12 | Q18 | Q24 | Q5 | Q11 | Q17 | Q23 | Q4 | Q10 | Q16 | Q22 | Q3 | Q9 | Q15 | Q21 | Q2 | Q8 | Q14 | Q20 | Q1 | Q7 | Q13 | Q19 |
| Q6/INT1 | **0.90** | 0.59 | 0.63 | 0.45 | 0.37 | 0.37 | 0.15 | 0.13 | 0.32 | 0.25 | 0.07 | 0.19 | 0.09 | 0.03 | -0.04 | 0.06 | 0.02 | 0.09 | -0.02 | 0.13 | -0.04 | 0.10 | -0.03 | -0.09 |
| Q12/INT2 | 0.47 | **1.07** | 0.81 | 0.74 | 0.38 | 0.39 | 0.25 | 0.24 | 0.16 | 0.23 | -0.02 | 0.26 | -0.01 | 0.12 | -0.14 | -0.05 | -0.04 | 0.07 | -0.09 | 0.06 | -0.03 | 0.05 | -0.11 | -0.09 |
| Q18/INT3 | 0.53 | 0.59 | **1.17** | 0.78 | 0.41 | 0.36 | 0.22 | 0.25 | 0.20 | 0.23 | -0.01 | 0.22 | -0.05 | 0.07 | -0.18 | -0.05 | -0.07 | 0.03 | -0.11 | 0.04 | -0.14 | -0.01 | -0.05 | -0.19 |
| Q24/INT4 | 0.46 | 0.60 | 0.70 | **1.65** | 0.32 | 0.46 | 0.55 | 0.50 | 0.23 | 0.41 | 0.11 | 0.46 | 0.11 | 0.22 | -0.02 | -0.03 | 0.04 | 0.17 | 0.04 | 0.11 | 0.10 | 0.08 | 0.08 | -0.04 |
| Q5/IDENT1 | 0.30 | 0.25 | 0.30 | 0.26 | **0.81** | 0.14 | -0.02 | 0.00 | -0.05 | -0.05 | -0.12 | -0.03 | -0.17 | -0.08 | -0.20 | -0.25 | -0.16 | -0.13 | -0.23 | -0.18 | -0.12 | -0.05 | -0.13 | -0.20 |
| Q11/IDENT2 | 0.37 | 0.53 | 0.51 | 0.78 | 0.12 | **1.32** | 0.40 | 0.33 | 0.45 | 0.47 | 0.20 | 0.47 | 0.18 | 0.19 | 0.13 | 0.15 | 0.12 | 0.16 | 0.15 | 0.20 | 0.12 | 0.16 | 0.06 | 0.08 |
| Q17/IDENT3 | 0.26 | 0.31 | 0.35 | 0.71 | 0.03 | 0.97 | **0.99** | 0.43 | 0.33 | 0.32 | 0.30 | 0.50 | 0.24 | 0.34 | 0.16 | 0.21 | 0.20 | 0.23 | 0.19 | 0.31 | 0.19 | 0.13 | 0.12 | 0.15 |
| Q23/IDENT4 | 0.29 | 0.30 | 0.31 | 0.74 | 0.05 | 0.72 | 0.84 | **0.73** | 0.29 | 0.25 | 0.23 | 0.44 | 0.25 | 0.24 | 0.16 | 0.19 | 0.18 | 0.18 | 0.21 | 0.32 | 0.12 | 0.12 | 0.13 | 0.09 |
| Q4/POSREG1 | 0.43 | 0.20 | 0.24 | 0.40 | 0.16 | 0.64 | 0.72 | 0.58 | **1.32** | 0.82 | 0.50 | 0.79 | 0.67 | 0.37 | 0.31 | 0.69 | 0.42 | 0.48 | 0.35 | 0.67 | 0.21 | 0.32 | 0.21 | 0.29 |
| Q10/POSREG2 | 0.26 | 0.11 | 0.10 | 0.34 | -0.02 | 0.60 | 0.67 | 0.55 | 1.29 | **1.22** | 0.41 | 0.83 | 0.54 | 0.45 | 0.29 | 0.57 | 0.30 | 0.38 | 0.36 | 0.58 | 0.15 | 0.19 | 0.18 | 0.16 |
| Q16/POSREG3 | 0.08 | 0.02 | 0.02 | 0.30 | -0.09 | 0.38 | 0.68 | 0.54 | 0.66 | 0.68 | **0.64** | 0.44 | 0.35 | 0.30 | 0.41 | 0.40 | 0.33 | 0.34 | 0.39 | 0.49 | 0.19 | 0.30 | 0.19 | 0.28 |
| Q22/POSREG4 | 0.27 | 0.14 | 0.15 | 0.33 | 0.01 | 0.48 | 0.66 | 0.65 | 1.30 | 1.21 | 0.70 | **1.20** | 0.53 | 0.50 | 0.30 | 0.57 | 0.28 | 0.34 | 0.31 | 0.68 | 0.17 | 0.20 | 0.20 | 0.20 |
| Q3/NEGREG1 | 0.23 | 0.06 | 0.07 | 0.10 | -0.07 | 0.46 | 0.58 | 0.59 | 1.08 | 0.90 | 0.54 | 0.92 | **0.95** | 0.36 | 0.46 | 0.65 | 0.40 | 0.45 | 0.47 | 0.56 | 0.20 | 0.24 | 0.25 | 0.33 |
| Q9/NEGREG2 | 0.12 | 0.01 | 0.02 | 0.21 | -0.06 | 0.30 | 0.47 | 0.42 | 0.71 | 0.85 | 0.59 | 0.90 | 0.67 | **0.57** | 0.25 | 0.31 | 0.23 | 0.24 | 0.26 | 0.31 | 0.19 | 0.24 | 0.20 | 0.25 |
| Q15/NEGREG3 | -0.02 | -0.08 | -0.12 | 0.04 | -0.15 | 0.19 | 0.35 | 0.26 | 0.47 | 0.50 | 0.59 | 0.52 | 0.70 | 0.45 | **0.61** | 0.50 | 0.28 | 0.36 | 0.51 | 0.49 | 0.26 | 0.26 | 0.24 | 0.36 |
| Q21/NEGREG4 | 0.20 | -0.01 | 0.04 | 0.05 | -0.06 | 0.38 | 0.54 | 0.52 | 1.00 | 0.85 | 0.60 | 0.93 | 1.35 | 0.59 | 0.84 | **1.18** | 0.37 | 0.48 | 0.58 | 0.87 | 0.21 | 0.30 | 0.32 | 0.40 |
| Q2/EXTREG1 | 0.02 | 0.02 | -0.07 | 0.20 | -0.21 | 0.21 | 0.38 | 0.38 | 0.49 | 0.49 | 0.59 | 0.49 | 0.58 | 0.38 | 0.51 | 0.52 | **0.54** | 0.27 | 0.34 | 0.37 | 0.26 | 0.24 | 0.23 | 0.33 |
| Q8/EXTREG2 | 0.01 | -0.04 | -0.09 | 0.03 | -0.11 | 0.17 | 0.07 | 0.18 | 0.21 | 0.22 | 0.30 | 0.22 | 0.43 | 0.36 | 0.40 | 0.40 | 0.35 | **1.06** | 0.49 | 0.39 | 0.30 | 0.37 | 0.24 | 0.25 |
| Q14/EXTREG3 | -0.01 | -0.04 | -0.05 | 0.06 | -0.10 | 0.12 | 0.23 | 0.31 | 0.31 | 0.34 | 0.48 | 0.36 | 0.56 | 0.38 | 0.71 | 0.71 | 0.48 | 0.53 | **0.83** | 0.62 | 0.27 | 0.29 | 0.32 | 0.42 |
| Q20/EXTREG4 | 0.17 | -0.04 | -0.04 | 0.19 | -0.05 | 0.31 | 0.43 | 0.51 | 0.80 | 0.77 | 0.68 | 0.80 | 0.76 | 0.54 | 0.73 | 0.98 | 0.64 | 0.43 | 0.69 | **1.33** | 0.33 | 0.37 | 0.31 | 0.47 |
| Q1/AMOT1 | -0.09 | 0.01 | -0.11 | 0.18 | -0.13 | 0.01 | 0.25 | 0.32 | 0.34 | 0.32 | 0.31 | 0.45 | 0.27 | 0.32 | 0.25 | 0.24 | 0.31 | 0.17 | 0.26 | 0.31 | **1.13** | 0.42 | 0.36 | 0.50 |
| Q7/AMOT2 | -0.22 | -0.12 | -0.08 | 0.02 | -0.17 | -0.02 | 0.09 | 0.18 | 0.27 | 0.39 | 0.34 | 0.47 | 0.36 | 0.40 | 0.36 | 0.48 | 0.35 | 0.28 | 0.47 | 0.42 | 0.47 | **1.17** | 0.31 | 0.63 |
| Q13/AMOT3 | -0.06 | -0.02 | -0.11 | 0.11 | -0.14 | 0.16 | 0.30 | 0.32 | 0.30 | 0.40 | 0.37 | 0.40 | 0.31 | 0.39 | 0.27 | 0.31 | 0.31 | 0.22 | 0.34 | 0.36 | 0.58 | 0.52 | **0.54** | 0.39 |
| Q19/AMOT4 | -0.03 | -0.07 | -0.10 | 0.12 | -0.10 | 0.02 | 0.24 | 0.35 | 0.42 | 0.37 | 0.41 | 0.53 | 0.52 | 0.48 | 0.49 | 0.59 | 0.39 | 0.27 | 0.58 | 0.75 | 0.55 | 0.75 | 0.64 | **0.97** |
| Notes: INT = intrinsic regulation; IDENT = identified regulation; POSREG = positive introjected regulation; NEGREG = negative introjected regulation; EXTREG = external regulation; AMOT = amotivation. ^a^ Sample 1 covariances are above the diagonal and variances are listed on the diagonal **(bolded values)**. Sample 2 covariances are below the diagonal. Sample 2 variances are: Q6 – 0.87, Q12 – 0.96, Q18 – 0.96, Q24 – 1.77, Q5 – 0.77, Q11 – 1.93, Q17 – 1.62, Q23 – 1.34, Q4 – 2.00, Q10 – 1.72, Q16 – 1.08, Q22 – 1.66, Q3 – 1.70, Q9 – 1.20, Q15 – 0.99, Q21 – 1.86, Q2 – 0.93, Q8 – 1.10, Q14, - 1.08, Q20 – 1.62, Q1 – 1.31, Q7 – 1.44, Q13 – 0.83, Q19 – 1.35. | | | | | | | | | | | | | | | | | | | | | | | | |
